# Alignment analysis of Brainlab knee 3 navigation-guided total knee arthroplasty using the adjusted mechanical method

**DOI:** 10.3389/fsurg.2022.1040025

**Published:** 2022-11-08

**Authors:** Yuqi Pan, Bowei Jiang, Yige Li, Yuhao Yu, Yunsu Chen

**Affiliations:** ^1^ Department of Joint Surgery, Shanghai Sixth People’s Hospital Affiliated to Shanghai Jiao Tong University School of Medicine, Shanghai, China; ^2^ Department of Rehabilitation, The Second Affiliated Hospital of Nanjing Medical University, Nanjing, China

**Keywords:** navigation TKA, AMA alignment, soft tissue balance, lower limbs alignment, brainlab knee3

## Abstract

**Purpose:**

With the application of navigation technology in Total Knee Arthroplasty (TKA), TKA procedures have become various. Studies have shown that navigation can improve the alignment of patients' lower limbs. To verify this conclusion, we collected the clinical data from patients who underwent Brainlab knee 3 navigation-guided TKA. Brainlab knee 3 is a completely new software that takes a different approach to address the current challenges of navigated TKA. During the procedure, we applied the Adjusted Mechanical Alignment (AMA) principle and took soft tissue balance as a priority. We aim to explore the patients’ lower limb alignment changes who underwent the Brainlab knee 3 navigation-guided TKA using the AMA method.

**Methods:**

Fifty consecutive patients who underwent total knee arthroplasty using the Brainlab knee3 knee navigation system (Smith&Nephew) from January to August 2021 by the same doctor (Yunsu Chen) in the Department of Joint Surgery of the Shanghai Sixth People's Hospital were included. Hip-Knee-Ankle Angle (HKAA), anatomic Femur Tibia Angle (FTA), Lateral Distal Femoral Angle (LDFA), and medial proximal tibia angle (MPTA) were measured on preoperative and postoperative full-length lower-limbs x-ray films or weight-bearing anterior and lateral knee radiographs for observational and descriptive study. The preoperative and postoperative knee alignment changes were analyzed through paired *t*-test or nonparametric Wilcoxon test using SPSS 25.0 software

**Results:**

Pre-operative and post-operative HKAA both obeyed normal distribution. The mean preoperative HKAA was 169.8° (154.9–178.7°) with a standard deviation of 5.41; the postoperative HKAA was 175.7° (168.4–180.0°) with a standard deviation of 2.81. Using the two-sample paired *t*-test to analyze, the result showed *P* = 0.000 < 0.05; a statistically significant difference exists. The preoperative and postoperative FTA obeyed normal distribution as well. The mean preoperative FTA was 174.7° (163.4–179.9°) with a standard deviation of 3.90; postoperative 175.6° (167.0–179.9°) with a standard deviation of 2.77. Using the two-sample paired *t*-test to analyze, the result showed *P* = 0.140 > 0.05, the difference was not statistically significant. The preoperative LDFA was normally distributed, while postoperative LDFA was not. The mean preoperative LDFA was 90.7° (83.5–99.6°) with a standard deviation of 3.83; the median of postoperative LDFA was 91.6° (86.0–103.2°) with an interquartile range of 2.93. Using the two-sample paired Wilcoxon test, the result showed *P* = 0.052 > 0.05; the difference was not statistically significant. Preoperative MPTA obeyed normal distribution, while postoperative MPTA did not. The mean preoperative MPTA was 83.5° (72.7–92.9°), with a standard deviation of 3.66; the median of postoperative MPTA was 89.3° (84.6–95.6°), with an interquartile range of 1.45. Using the two-sample paired Wilcoxon test, the result shows *P* = 0.000 < 0.05; a statistically significant difference exists.

**Conclusion:**

In our study, AMA alignment was applied in Brainlab Knee3 computer navigation-assisted total knee arthroplasty. The femoral and tibial osteotomy angles were minimally adjusted according to soft tissue situations to reduce soft tissue release. We found AMA alignment provides good control of knee alignment in the coronal plane of the lower limbs, which is a reliable technique.

## Introduction

As a mature surgical technique, total knee arthroplasty (TKA) can effectively treat end-stage knee osteoarthritis, rheumatoid knee arthritis, traumatic knee arthritis, and other knee diseases, improving patients' quality of life. However, about 11%–25% of patients are not satisfied with the clinical outcomes after TKA (1). In recent years, with the rapid development of artificial intelligence, computer navigation technology has been applied in TKA to improve the accuracy of prosthesis positions. Compared with traditional manual surgery, it significantly improves the lower limb alignment, reduces the wear and loosening rate of the prosthesis, increases the prosthesis survival rate, and accelerates the postoperative rehabilitation process (2–4).

Navigation-guided TKA allows surgeons to quantitatively check the patient's current disease status in real-time, using knee flexion/extension or varus/valgus movements to assess knee parameters. Surgeons can more easily balance knee flexion and extension gap as well as manage soft tissue situations. After registration of the anatomic landmarks by the pointer, the knee navigation system establishes a simulated knee joint 3D image on the screen, showing the alignment and stability condition, and further predicting the appropriate position of the osteotomy direction and the geometric shape of the prosthesis. Compared with traditional surgical techniques, navigation systems can provide better alignment and lower outliers, reducing the revision rate (5). The clinical outcomes of the navigation remain unsure. There is research showing similar clinical outcomes between navigation TKA and other procedures (6, 7).

The Brainlab Knee3 is a brand-new system that solves many current problems of navigation-guided TKA. KNEE3 is a smart imageless navigation software from Brainlab that visualizes and summarizes the complex interaction between 3D-kinematics, joint stability and implant alignment. The knee navigation application is designed to seamlessly fit into the surgeon's preferred technique, allowing them to quickly assess cutting block position. Without the need to touch the monitor during surgery, KNEE3 simplifies soft tissue management. The Knee3 dynamically shows the stability of the knee with different ranges of motion so that the surgeon can estimate the kinematic parameters of the knee even before the osteotomy step. The potential outcomes of the surgical procedure and real-time results are fed back to the surgeon for judgment and make the corresponding adjustment.

Since the introduction of the Smith & Nephew Navigation &Brainlab Knee 3 system in the Joint Department of Shanghai Sixth People's Hospital, a large number of navigation-guided total knee replacement surgeries have been performed. We collected the data of patients who underwent total knee arthroplasty with the Smith & Nephew Brainlab KNEE3 knee navigation system from January to August 2021 by a single doctor (Yunsu Chen) at the Joint Surgery Department of Shanghai Sixth People's Hospital and evaluated their imaging data. The lower limb alignment was measured by preoperative and postoperative full-length x-ray or anterior/posterior and lateral view film of the knee joint. The study aims to explore the patients' lower limb alignment changes who underwent the Brainlab knee3 navigation-guided TKA using the AMA method.

## Materials and methods

This study has been permitted by the ethics committee of Shanghai sixth people's hospital. (Ethics number: YS - 2018-103). All patients agreed on data collection and analysis by written consent. Inclusion criteria: (1) primary total knee arthroplasty; (2) pre-operation and post-operation radiographic information is complete; (3) using Brainlab knee 3 navigation system and posterior–stabilized prosthesis(PS) for posterior cruciate ligament resection. Exclusion criteria: (1) preoperative extra-articular deformity; (2) preoperative knee valgus; (3) recent knee infection and the surgery or trauma history of the knee joint.

61 patients’ data were collected. After 11 patients who don't meet the criteria (2 patients with valgus knee, and 9 patients with incomplete imaging files) were ruled out, a total of 50 patients were included in the study, including 18 male patients and 32 female patients. The average age was 72.3 (56–85) years old. There were 29 cases with left knee TKA and 21 cases with right knee TKA. All patients were asked to take a weight-bearing anterior/posterior-lateral view of the knee joint and a full-length x-ray of the lower limbs.

### Surgical procedure

After the camera and screen were well-placed, open the navigation system. The patient was under general anesthesia. Routinely disinfect and pave sterile sheets for the patient. Bone reference arrays and bone fixators were assembled and installed to stabilize the lower limbs. Assemble the registration probe. Using parapatellar path to expose the knee joint. Assess the extent of arthritic damage and the ligaments and remove all prominent osteophytes from the medial (or lateral) edges of the femur and tibia and in the intercondylar notch as they may affect soft tissue balancing. Two 3.2 mm double cortical tibia pins were placed in the mid-shaft on the anterior tibia, and a tibia reference array was installed. Register a series of anatomical landmarks as instructed by the navigation system. On the femur, anatomical markers such as femoral head center, distal femoral axis point, medial and lateral epicondyles, transepicondyle axis, Whiteside line, and anterior femoral cortex were successively registered. The acquisition of the femoral head center along with the distal end of the femur defines the femur mechanical axis to determine the inversion/eversion, flexion/extension of the femoral prosthesis, and the lower limbs alignment. The medial and lateral epicondyle points define the transepicondyle axis which represents the anteroposterior orientation of the femur. The transepicondylar axis and Whiteside line can be used as a reference for the rotation alignment of the femoral component. The femoral condyle was registered by obtaining several points along the surface of the medial and lateral condyles with the pointer. Multiple points along the anterior cortical surface were obtained to register the anterior femoral condylar cortex.

On the tibia, the following anatomic markers were registered: medial and lateral malleolus, proximal tibial mechanical axis point, tibia A/P direction, and medial and lateral tibial plateau. The proximal point on the tibial mechanical axis was obtained by registering the posterior aspect of the anterior cruciate ligament (ACL) insertion point. The pointer is placed horizontally in the anterior-posterior (AP) direction so that it lies on the tibial eminence, and the tibial AP direction is registered. Register several points on the tibial plateau.

After registration, the screen displays the current flexion/ extension and varus/valgus alignment of the limb as well as measurements regarding the implant position and expected stability.

Bring the leg into full extension and apply varus and valgus stress to test stability and check for fixed flexion or other deformities. Bring the leg into maximum flexion applying varus and valgus stress again during the movement.

Evaluate femoral implant size. The plan for the femoral component is based on the implant specifications following a measured resection philosophy. Femoral sizing is based on the AP dimensions of the registered femur. Resection height values are based on the registered femoral landmarks. Ensure that the prosthesis used is the same size as the planned prosthesis, otherwise, adjust accordingly. Apply the AMA principle for the osteotomy step, allowing 3° varus/valgus for the lower limb alignment.

#### Distal femoral osteotomy

The cutting block is placed on the distal femur. Potential inequality of extension and flexion gap can be addressed by altering the cutting block position. The extension gap is reduced or increased by moving the osteotomy plate distal or proximal. The flexion gap can be decreased by slightly flexing the distal cut and therefore the femoral component. To increase the flexion gap, consider reducing the size of the femoral prosthesis. The asymmetry of the extension gap can be solved by removing osteophytes or releasing soft tissue. After determining the position of the cutting block, fixation was performed. After the resection has been performed, verify the cut using the plane tool with the verification plate.

#### Anterior and posterior femoral osteotomy

At this point, the flexion gap can be adjusted to match the extension gap. The flexion gap was increased/decreased by adjusting the cutting block positions anteriorly or posteriorly. If the flexion gap is larger than the extension gap, consider reducing the femoral implant size. Increase the size instead if the flexion gap is smaller than the extension gap.

For a given medial/lateral flexion gap mismatch, there are usually three options to achieve a balanced gap: (1) adjust femoral component rotation: internal rotation of the cutting block will close the medial and open the lateral flexion space, while external rotation will open the medial and close the lateral compartment of the joint in flexion. Rotate the cutting block until medial and lateral flexion gaps are equalized. (2) Perform additional soft tissue management: an imbalance can be a sign of an incomplete release. Make sure to remove all osteophytes. Always consider the effect of the particular release for the extension gap as well to avoid over-releasing. (3) Allow for a range of instability: natural knees commonly tend to have a laxer lateral compartment, particularly in flexion. Slight lateral instability is acceptable to avoid excessive rotation of the implant.

Then, the femoral intercondylar osteotomy was performed. Validate the incision. Check the knee stability updated by the system in real-time.

#### Tibial osteotomy

Both the extension and the flexion gap will open or close when distalizing or proximalizing the tibia resection. Once the desired cutting block position is achieved, pin the block with the first pin, fixing the tibia slope angle. Adjust the varus/valgus position to achieve gap symmetry and fix the block with the second fixation pin. Similarly, trim and clean the tibial osteotomy surface and verify the resection.

**Figure 1:**
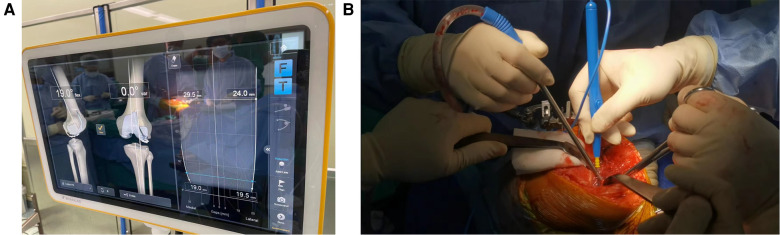
Surgical method sketch. (**A**) The Brainlab knee3 system shows the alignment and stability of the lower limbs in surgery. (**B**) The soft tissue release on the medial side of the knee.

At full extension, the medial and lateral knee gap symmetry and stability are examined. If there is asymmetry, consider additional osteophyte removal or soft tissue release. For medial soft tissue management, if it is necessary, release the medial soft tissue layer by layer until the deep part of the medial collateral ligament (MCL), 1 cm–1.5 cm away from the joint line. The effect of releases can immediately be visualized by applying medial or lateral stress in a certain flexion range. After the insertion of the trial components and the implant, the capsule was sutured and the incision was closed. Figure 1 shows the Brainlab knee3 navigation system and the soft tissue release in surgery.

### Data collection and analysis

**Figure 2:**
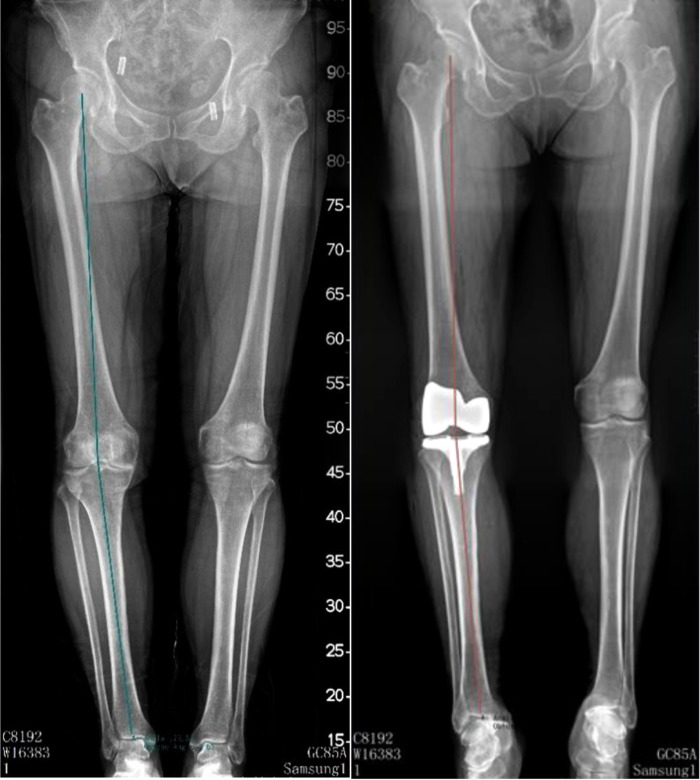
Pre-operation and post-operation HKAA measurement method.

**Figure 3:**
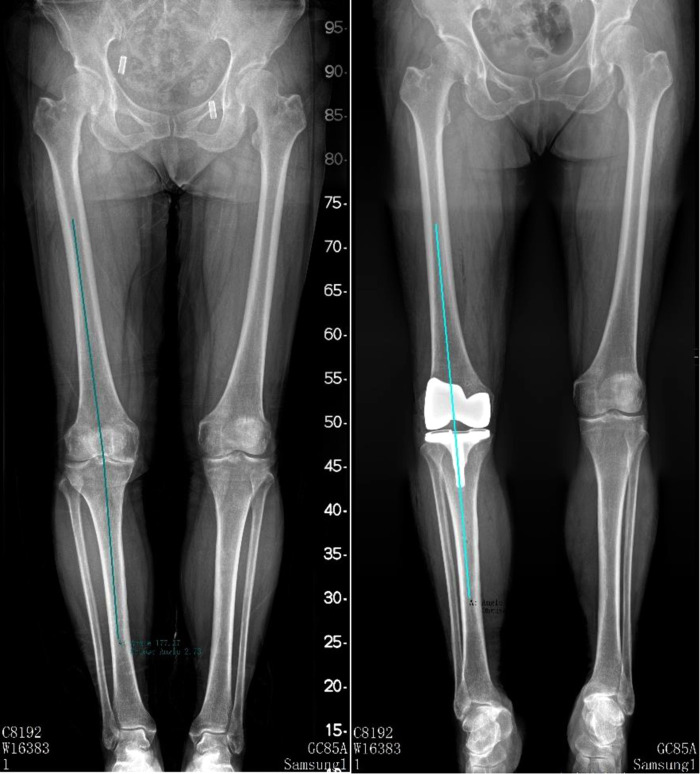
Pre-operation and post-operation FTA measurement method.

**Figure 4:**
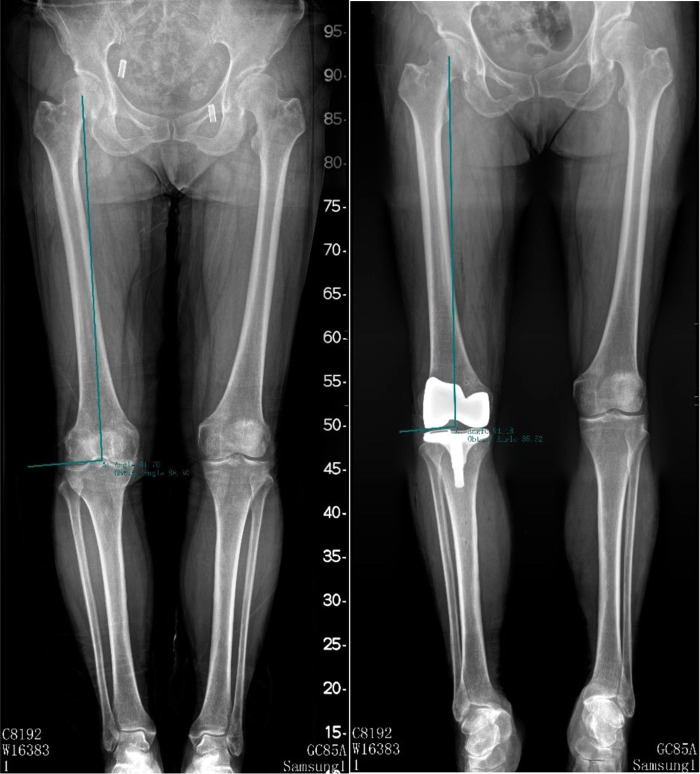
Pre-operation and post-operation LDFA measurement method.

**Figure 5:**
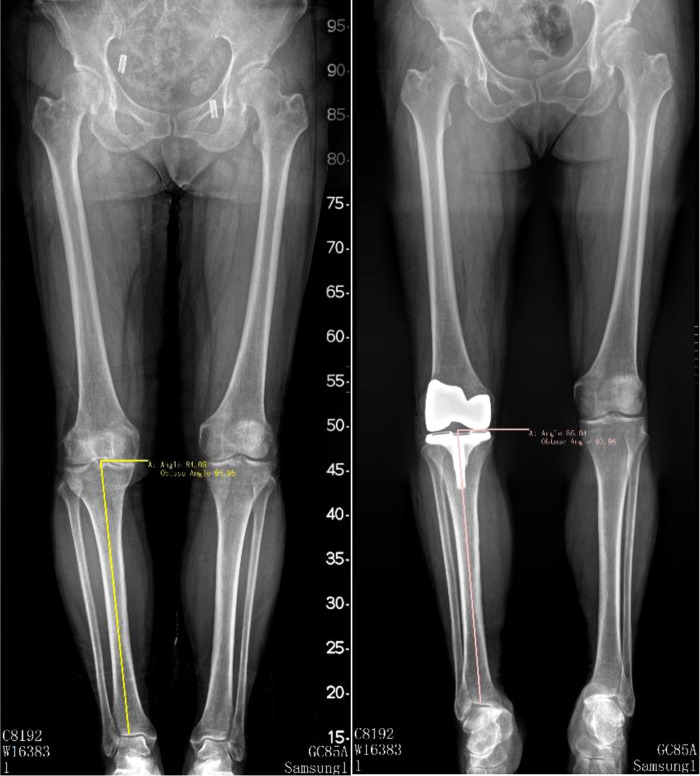
Pre-operation and post-operation MPTA measurement method.

Hip-knee-ankle angle (HKAA), anatomic femur tibia angle (FTA), lateral distal femoral angle (LDFA), and medial proximal tibia angle (MPTA) were measured on full-length lower-limbs x-ray films or weight-bearing anterior and lateral knee radiographs preoperatively and postoperatively (3 months after surgery). HKAA is the angle between the femoral mechanical axis and the tibial mechanical axis (see Figure 2). FTA is the angle between the femur anatomical axis and the tibia anatomical axis (see Figure 3). LDFA is the lateral angle between the femoral mechanical axis and the tangent of the medial and lateral femoral condyle (see Figure 4). MPTA is the medial angle between the tibial mechanical axis and the tangent of the tibial plateau (see Figure 5). All measurements were performed by the same doctor from the joint surgery department. Each angle was measured three times and averaged to one decimal place. The results were reviewed by another senior surgeon from the joint surgery department.

### Statistical analysis

SPSS25.0 statistical software was used for analysis. Since the sample size was 50, the Shapiro–Wilk test (S–W test) was used to test whether the samples obeyed normal distribution. When the measurement data followed normal distribution, the mean and standard deviation were described. The comparison of pre-operation and post-operation results was performed by paired two-sample *t*-test. When normal distribution was not met, the median and interquartile distance were described. The paired two-sample Wilcoxon rank sum was used for pre-operation and post-operation comparison.

## Results

### HKAA

The *P* value of the S–W test for pre-operation and post-operation HKA were both >0.05, following the normal distribution. The mean preoperative HKA was 169.8° (154.9–178.7°), the standard deviation was 5.41, and there were 3 cases in the range of 180 ± 3°. Postoperative 175.7° (168.4–180.0°), standard deviation 2.81, a total of 16 cases in the range of 180 ± 3°. Using the two-sample paired *t*-test to analyze, *t* = −8.339, *P* = 0.003 < 0.05, the difference was statistically significant (see Table 1).

**Table 1:** Pre-operation and post-operation HKAA comparison.

	Min	Max	Mean	SD	180 ± 3°samples	*P* (S–W test)	*P* (*t*-test)	95%CI
Pre-op HKAA (°)	154.9	178.7	169.8	5.4	3 (6%)	0.169	0.003	−7.27, −4.44
Post-op HKAA (°)	168.4	180.0	175.7	2.8	16 (32%)	0.053		

*Min refers to minimum, Max refers to maximum, SD refers to standard deviation, CI refers to confidence interval, Pre-op refers to pre-operation, Post-op refers to post-operation.

### FTA

The *P* value of the S–W test for pre-operation and post-operation FTA were both >0.05, following the normal distribution. The mean preoperative FTA was 174.7° (163.4–179.9°) with a standard deviation of 3.90. Postoperative 175.6° (167.0–179.9°), standard deviation 2.77. Using two-sample paired *t*-test analysis, *t* = −1.500, *P* = 0.140 > 0.05, the difference was not statistically significant (see Table 2).

**Table 2:** Pre-operation and post-operation FTA comparison.

	Min	Max	Mean	SD	*P* (S–W test)	*P* (*t*-test)	95%CI
Pre-op FTA (°)	163.4	179.9	174.7	3.90	0.297	0.140	−2.23, 0.32
Post-op FTA (°)	167.0	179.9	175.6	2.77	0.136		

*Min refers to minimum, Max refers to maximum, SD refers to standard deviation, CI refers to confidence interval, Pre-op refers to pre-operation, Post-op refers to post-operation.

### LDFA

The preoperative S–W test *P* value of LDFA was > 0.05, which followed the normal distribution. Post-operation *P* < 0.05, did not follow the normal distribution. The mean preoperative LDFA was 90.7° (83.5–99.6°); the standard deviation was 3.83, and 26 cases were in the range of 90 ± 3°. The median of post-operation LDFA was 91.6; the interquartile range was 2.93, and 33 cases were within 90 ± 3°. The two-sample paired Wilcoxon test was used for analysis, *Z* = −1.945, *P* = 0.052 > 0.05, and the difference was not statistically significant (see Table 3).

**Table 3:** Pre-operation and post-operation LDFA comparison.

	Min	Max	Mean/Median	SD/	90 ± 3°samples	*P* (S–W test)	*P* (Wilcoxon-test)
Pre-op LDFA (°)	83.5	99.6	90.7	3.83	26 (52%)	0.483	0.052
Post-op LDFA (°)	86.0	103.2	91.6	2.93	33 (66%)	0.005	

*Min refers to minimum, Max refers to maximum, SD refers to standard deviation, IQR refers to interquartile range, Pre-op refers to pre-operation, Post-op refers to post-operation.

### MPTA

The preoperative S–W test *P* value of MPTA was >0.05, which followed the normal distribution. Post-operation *P* < 0.05, did not follow the normal distribution. The mean preoperative MPTA was 83.5° (72.7–92.9°); the standard deviation was 3.66, and 7 cases were in the range of 90 ± 3°. The median of post-operation MPTA was 89.3° (84.6–95.6°); the interquartile range was 1.45, and 47 cases were within 90 ± 3°. The two-sample paired Wilcoxon test was used for analysis, *Z* = −5.749, *P* = 0.000 < 0.05, the difference was statistically significant (see Table 4).

**Table 4:** Pre-operation and post-operation MPTA comparison.

	Min	Max	Mean/Median	SD/IQR	90 ± 3°samples	*P* (S–W test)	*P* (Wilcoxon-test)
Pre-op MPTA (°)	72.7	92.9	83.5	3.66	7 (14%)	0.179	0.000
Post-op MPTA (°)	84.6	95.6	89.3	1.45	47 (94%)	0.000	

*Min refers to minimum, Max refers to maximum, SD refers to standard deviation, IQR refers to interquartile range, Pre-op refers to pre-operation, Post-op refers to post-operation.

## Discuss

The lower limbs alignment adjustment and soft tissue balance are the key points in TKA. The goal of TKA surgery is to equalize the flexion and extension gaps with proper osteotomy and soft tissue release. The application of navigation helps us to perform more accurate osteotomy with computer assistance, achieving the ideal alignment and minimizing soft tissue release, to pursue more satisfactory results.

### Alignment analysis

At present, the standard of TKA alignment is still controversial. The goal of most TKA procedures is to correct the mechanical axis of the lower limbs to a neutral 180° position and set the femoral and tibial prosthesis perpendicular to the mechanical axis of the femur and tibia respectively, known as mechanical alignment (MA). For decades, a stable knee with a neutrally aligned lower limb has been one of the primary goals of TKA because it was supposed to be important for successful clinical outcomes and implant survivorship (8). When using manual instruments, the tibial extramedullary guide is usually placed 90° perpendicular to the tibial mechanical axis. The femur is prepared with an anatomic valgus cut (typically 5°–7°), resulting in an overall lower limbs mechanical axis alignment of 180° (9).

Considering that most people's knee joint line is not parallel to the ground, but has a constitutional varus of about 3°, it is questionable to set the absolute neutrality of the lower limbs as the goal of all TKA operations (10). As a result, a modified MA technology has emerged. AMA (Adjusted Mechanical Alignment) minimizes soft tissue release by placing the femoral implant in a slightly varus position. It is a modification of the classic Mechanical Axial Alignment (MA), which minimizes soft tissue release by adjusting the distal femoral osteotomy according to individual extension gap differences, taking the knee's natural ligament tension into account. It allows the HKAA to have a varus deviation within 3° (11), which is more consistent with the physiological knee alignment. Mild to moderate deformities of lower limbs were retained, and more severe deformities were corrected (8).

Besides MA Alignment and AMA Alignment principle, another alignment method is kinematic alignment (KA). The goal of KA is to alter the knee's physiological biomechanics as little as possible by restoring native (pre-arthritic) knee joint line alignment and ligament laxities. KA refers to the articular surface condition, compensates for cartilage/bone loss, and considers the thickness of the implants to set up the orientation and height of osteotomy. In this way, the implant can be aligned on the kinematic axis of the knee joint (12). However, the use of KA in the treatment of knee with moderate and severe varus may lead to uneven force on the knee joint, increased the contact force, stress and bone strain at the medial side, raising the risk of tibial plateau collapse and prosthesis wear/loosening rate (13).

There are various methods of alignment in TKA, and the research conclusions are not the same. While some studies demonstrated an increased revision risk in malaligned TKA, other studies have pointed out that there was no difference between TKA with a mechanical axis within or outside 0 ± 3° (14–16).

To release the soft tissue as less as possible, we performed the surgery using the AMA principle with soft tissue balance as the priority. The average postoperative HKAA Angle was 175.7°, close to the deviation range of 3°, which was statistically significant compared with pre-operation. Among them, 16 cases were within 180 ± 3° after the operation, which was significantly improved compared with 3 cases before the operation. Relevant literature has reported and analyzed the superiority of navigation over traditional surgery (17) and patient specific instrument (PSI) (18, 19) to achieve the required HKAA. A retrospective study of 600 patients undergoing navigational knee replacement surgery showed that 91% of patients' HKAA could achieve 180 ± 3° after surgery, with 90% of patients using Brainlab navigation (20).

FTA is the Angle between the anatomical axis of the femur and tibia, which can be used to measure the lower limb's coronal alignment (21). Some studies have discovered there were some correlations between FTA and lower limb HKAA (22). FTA has a 5–7° valgus angle relative to the lower limb's mechanical axis (23). In this study, the mean postoperative FTA was 175.6°, which was not statistically different compared to preoperative FTA. The reason may be that the anatomical relationship of the femur and tibia was only adjusted minimally during the operation, and the change of the angle before and after the operation was not as obvious as that of HKAA.

LDFA and MPTA represent the varus and valgus deviation of the femur and tibia relative to the lower limb's mechanical axis in the coronal position and the alignment after knee reconstruction (24). Generally speaking, LDFA decreases in knee valgus, while MPTA decreases in knee varus (25, 26). Studies have reported that more than 3° coronal malalignment of the prosthesis in TKA may increase the risk of aseptic loosening. Osteotomy of the distal femur and proximal tibia should be perpendicular to their respective mechanical axes (27). In the ideal condition of the lower limb's mechanical alignment, LDFA and MPTA are both 90°. Considering the constitutional varus of the knee joint at 3° in most people, a slight varus MPTA between 87° and 88° was shown to be more physiologic (3). In our study, there was no statistically significant difference between postoperative LDFA and preoperational LDFA, while there was a statistically significant difference between postoperative MPTA and preoperational MPTA. The median postoperative value was 89.3°, of which 47 cases were within 90 ± 3° compared with only 3 cases before surgery. Postoperative MPTA is closer to the natural knee standard.

### Soft tissue balance

In TKA surgery, the appropriate soft tissue release is needed to create a symmetrical balance of flexion and extension gap (the difference is less than 3 mm) (28). The incidence of varus deformity is higher than valgus deformity of the knee joint, and more attention should be paid to medial soft tissue release (29).

According to the knee varus degree, conventional TKA gradually releases the superficial, deep MCL, and the pes anserinus. If necessary, the deep layer of the soleus muscle and the attachment of the semimembranosus muscle at the tibial epiphysis can also be released with a bone chisel, thus exposing the medial tibia. Although deep MCL release and osteophyte resection is routinely performed in TKA, the medial soft tissue should be released as less as possible because the excessive release of the medial soft tissue may lead to medial instability, mid-flexion instability, hematoma formation, knee joint elevation, and the need for constrained implants, which contribute to poor postoperative outcomes (30).

In our study, soft tissue was released up to the deep part of the MCL, 1–1.5 cm distal to the knee joint line. If the release was still not adequate, consider adjusting the femoral varus/valgus and rotation positions. Using AMA alignment to achieve less soft tissue release.

### Advantages of navigation in TKA

Computer navigation can significantly improve the alignment of the implant and lower limbs. A meta-analysis of 23 controlled randomized trials reported that at both the 3° and 2° threshold for malalignment from neutral in the mechanical axis, significantly fewer patients in the navigated arthroplasty group were outside of this value compared with the conventional TKA (31).

Moskal (32), Zhao (33) et al. also proved that navigation TKA could better correct the lower limb mechanical axis through meta-analysis. Mooney et al. (34) showed that navigation-enhanced instrumentation significantly reduced the total outlier rate (±2°/2 mm) as compared to conventional instrumentation. The superiority of computer navigation over conventional TKA in improving accuracy is well established (35). There is an abundance of evidence that computer navigation produces better precision than conventional instrumentation, but only limited evidence that this translates into better clinical outcomes (36). Although recent reviews revealed the superiority of the navigation-TKA technique over the conventional technique remains uncertain in the short and long term, the use of computer navigation TKA is an example of an initiative to augment human decision-making and surgical handicraft with artificial intelligence (37).

Smith & Nephew Brainlab Knee3 navigation system is a product promoted in China in recent years. It has the advantages of farsightedness, real-time feedback, and result visualization, which brings great convenience to TKA surgery. The alignment and stability of the knee can be assessed immediately after the registration of the corresponding anatomical markers by the surgeon. As the knee moved from full flexion to full extension, the screen analyzed the balance gap, and the stability of the knee was calculated according to the estimated implant geometry size before osteotomy. The system can help doctors identify potentially unstable or malformed conditions and intelligently display the next surgical steps. Take the tibial resection for example, without any assessment direction, the software displays the respected view and the cutting block can be placed. This patient-specific navigation allows the surgeon to foresee the outcome of final joint stability based on the implant and according to previously verified cut geometry. Intraoperative soft tissue balance is also taken into account. Finally, when performing the anterior femoral cut, the system demonstrated how the rotational cutting block will affect the final stability. The goal is to maintain two parallel osteotomy lines indicating the asymmetry ability of the knee joint. By selecting the appropriate prosthesis, the prosthesis also appears in the evaluation interface. The system can improve flexion and extension balance and lower limb alignment.

The Smith & Nephew Brainlab KNEE3 navigation system was applied in the TKA surgery. Soft tissue release was minimized by using AMA alignment. Post-operative alignment on full-limbs x-rays improved significantly compared with pre-operation.

### Severe preoperative joint deformity in navigation TKA

Severe knee varus deformity is often associated with medial soft tissue flexion contracture and lateral soft tissue elongation (38), while severe valgus knee may cause lateral femur and tibia bone defect, lateral soft tissue tightening, and medial soft-tissue laxity (39), making navigation TKA more difficult. A study compared 10-year clinical and radiographic outcomes and survival rates between navigation TKA and conventional TKA in patients with preoperative severe varus deformity. The results showed navigation TKA had fewer outliers in the HKA angle for severe preoperative varus deformity compared with conventional TKA. The long-term clinical outcomes and survival rates were similar between the two techniques (40). Another study compared three TKA surgical methods: conventional TKA、navigation TKA and the patient specific instrumentation, which were applied in the severe knee varus/valgus (>10°). 159 patients were included in the study and the author found that three surgical techniques demonstrated similar postoperative radiographic alignment (41). Researches about severe varus/valgus knee deformity in navigation TKA are not so many. Since our study only included patients who had mild varus knee deformity, further studies should be performed to explore the outcomes of severe joint deformity in Navigation TKA. Another challenging case is the extra-articular deformity. And in these situations, it's tricky to handle the lower limb's alignment. More navigation TKA procedures have been applied over the last couple of decades to treat the extra-articular deformity. Navigation is recommended for these challenging cases because of its accuracy (42).

## Research limitations

Firstly, the sample number was 50, which was not high enough. Secondly, the study only involved the evaluation of radiological alignment, without considering clinical outcomes. Thirdly, the study was a comparison between post-operation and pre-operation alignment, no control group was set up with manual total knee arthroplasty. The alignment measurement was subjective, and the x-ray films were followed up only 3 months after the operation. Despite these limitations, the study evaluate the alignment changes for the Brainlab knee 3 navigation system with the AMA principle, and the outcomes were meaningful.

## Conclusion

The alignment adjustment and soft tissue balance are always the key points in TKA. According to the concept of mechanical alignment, the tibial osteotomy is perpendicular to the tibial mechanical axis, and the femoral osteotomy is perpendicular to the femoral mechanical axis. Both LDFA and MPTA should ideally be close to 90 degrees. However, some studies have pointed out that the use of AMA osteotomy is more physiological. In this study, Brainlab Knee 3 navigation system and AMA alignment principle were used to perform TKA for patients, giving priority to the soft tissue balance during the operation. The general alignment after the operation was closer to the physiological standard than before the operation, and the fluctuation of results was small. In addition, we found that LDFA did not differ significantly before and after surgery, whereas MPTA showed significant differences before and after surgery, closer to the 90 ± 3° range. Further study should be launched to explore the underline reason.

Compared with manual surgery, the computer navigation system can dynamically evaluate the limb changes in the whole range of motion of the knee joint, provide the mechanical, anatomical, and kinematic alignment of the knee joint, as well as the flexion and extension space, and predict the results of different operations in real-time to the surgeon, to assist the surgeon to achieve better soft tissue balance.

Brainlab Knee3 computer navigation assisted total knee arthroplasty with intraoperative AMA alignment technique was used to fine-tune the osteotomy angle of the femur and tibia according to the soft tissue balance, to reduce soft tissue release. This study found that Brainlab Knee3 computer-guided TKA using AMA method can well control the postoperative lower limb alignment indicators, which is a reliable technique.

## Data Availability

The raw data supporting the conclusions of this article will be made available by the authors, without undue reservation.
